# An Intraocular Pressure Measurement Technique Based on Acoustic Radiation Force Using an Ultrasound Transducer: A Feasibility Study

**DOI:** 10.3390/s21051857

**Published:** 2021-03-07

**Authors:** Hee Su Lee, Eun Young Jeong, Jin Ho Sung, Bo Eun Choi, Jong Seob Jeong

**Affiliations:** Department of Medical Biotechnology, Dongguk University, Seoul 04620, Korea; 22sooooo@dongguk.edu (H.S.L.); chocoeun7@dongguk.edu (E.Y.J.); madeinjinho@dongguk.edu (J.H.S.); chbe0306@dongguk.edu (B.E.C.)

**Keywords:** acoustic radiation force, ultrasound transducer, intraocular pressure, tonometry, eye phantom

## Abstract

High intraocular pressure (IOP) is one of the major risk factors for glaucoma, and thus accurate IOP measurements should be performed to diagnose and treat glaucoma early. In this study, a novel technique for measuring the IOP based on acoustic radiation force was proposed, and its potential was experimentally demonstrated. The proposed technique uses the acoustic radiation force to generate axial displacement on the ocular surface while simultaneously measuring the degree of deformation. In order to verify that the ocular displacement induced by the acoustic radiation force is related to the IOP, the experiment was conducted by fabricating a 5 MHz single element transducer and gelatin phantoms with different stiffness values. Our experimental results show that there is a close relationship between the ocular displacement by the acoustic radiation force and the IOP obtained by a commercial tonometer. Therefore, the proposed acoustic radiation force technique can be a promising candidate for measuring the IOP.

## 1. Introduction

It is well known that glaucoma is one of the primary leading causes of blindness [1,2,3,4]. Without proper treatment, glaucoma can degenerate visual ability, and eventually result in an irreversible vision loss [2,3,4]. Although glaucoma is a multifactorial disease, increased intraocular pressure (IOP) is one of the major risk factors for its progression. Therefore, in many cases glaucoma can be treated by reducing the IOP [3,4,5,6,7,8]. The IOP is the fluid pressure of the eye, that is, the magnitude of the force generated by the aqueous humor on the internal surface area of the anterior eye [4]. Increasing the IOP can cause mechanical stress and ischemic effects on the retinal nerve fiber layer, thus, accurate IOP measurements are needed for efficient diagnosis to perceive the risk of developing glaucoma in advance as well as for assessment of progress after treatment.

Although there are several methods for measuring the IOP, Goldmann applanation tonometry (GAT) is known well as a classical gold standard method. The GAT determines the IOP from the force required to flatten a pre-defined area of the central cornea [9,10,11,12,13]. This assessment, however, has some limitations, requiring patients to maintain open the upper eyelid during measurement, necessity for anesthesia and fluorescein staining, direct contact with the cornea, immobility due to the slit-lamp set up, and the influence of the central corneal thickness (CCT) measurable with an additional instrument such as an ultrasound pachymeter [11,12,13,14].

To solve these problems, new methods for IOP measurement are being developed, especially, a rebound tonometer which uses an induction/impact principle [15]. It is based on launching a magnetized probe with a plastic end-tip towards the cornea and monitoring the voltage induced in a solenoid coil as it returns [11,12,15,16,17,18]. This instrument is portable and easy to use, and does not require topical anesthesia [11,12,18,19,20]. However, it causes patients discomfort and may inflict cornea injury since it is accomplished while they open their eyes, and the probe hits cornea directly. In addition, the CCT has influence on the measurement of the rebound tonometer as well as GAT, resulting in need for any assessment to always be accompanied by a pachymeter measurement [19,20,21].

Unlike contact tonometers, a non-contact tonometer (NCT) is free from mechanical contact with the eye, providing a non-invasive test and minimal risk of infection [13,22,23,24]. The NCT, introduced by Grolmann in 1972 [25], generates a puff of air, whose force increases linearly over time and collimates a beam of light. The cornea is flattened by the air puff and reflects a light beam onto a sensor. The time required to produce the peak reflection is measured and converted to the IOP [13,22,23,24,25,26]. Although there are several advantages such that the NCT does not require the use of anesthesia and can be operated by paramedical personnel because of its simple operating process, the air puff still startles patients, and the measured results are more affected by the CCT compared to the GAT. In addition, it has decreasing reliability at higher pressure ranges [22,27].

In the same vein, to measure the IOP using a non-contact method, this paper presents a novel mechanism to assess the IOP using acoustic radiation force. Acoustic radiation force has been widely used in elasticity imaging, visually providing elastic information of a tissue which is hard to identify using a conventional brightness mode (B-mode) image. This radiation force, produced by a burst of ultrasound waves, generates a localized tissue displacement that can be detected by using an ultrasound correlation-based method. The tissue displacement response is dependent on the magnitude of the applied force and inversely proportional to the tissue stiffness [28,29,30,31,32,33,34,35]. That is, when a certain force is applied, measuring the tissue displacement can provide information about its elasticity. If the ultrasound transmission intensity is within a safe range, it does not have any harmful effects on the human body, does not require anesthesia or direct contact with the cornea, and is not limited by the subject’s position or angle, thereby minimizing patient discomfort. A single transducer can generate an acoustic radiation force to produce a tissue response and track the resulting displacement at the same time.

There is another imaging technique that measures tissue displacement known as the optical coherence elastography (OCE) technique. In this scheme, a laser pulse, needle probe, or acoustic radiation force are used to produce an external stimulation to generate tissue displacement, and the resulting changes are measured by the optical coherence technique (OCT) [36,37,38,39,40]. However, OCE eventually requires two different energy sources for tissue displacement generation and measurement. Thus, a complicated probe and a system in which the external simulation source is integrated with the OCT are required [36]. In addition, to the best of our knowledge, the OCE has been studied for elastic imaging that presents the mechanical properties of corneal tissue, but it has not been applied to tonometry.

The purpose of this research is to demonstrate the correlation between the IOP and displacement generated by an acoustic radiation force, and verify the feasibility through experiments. The analysis of the correlation mechanism was performed by calculating a formula that converts the displacement into an IOP value. For displacement assessment, gelatin-based phantoms having different elasticity were fabricated by controlling the gelatin concentration. A pushing beam for generation of acoustic radiation force and tracking beams for detection of displacement were produced by a specially designed ultrasound beam sequence using a customized transducer having a 5 MHz center frequency. The radio frequency data was obtained and processed off-line to calculate the axial displacement along the beam axis. To demonstrate the performance of the proposed method, we compared the resulting displacement with the IOP obtained by a commercial rebound tonometry apparatus.

## 2. Materials and Methods

### 2.1. Acoustic Radiation Force

The acoustic radiation force is a phenomenon caused by a transfer of momentum from an acoustic wave to a propagation medium when the wave is absorbed or reflected by a target [28,29,30,31,41,42]. This force can be expressed by the following equation [28,29,30,42]:

(1) $$F=\frac{W_{absorbed}}{c}=\frac{2\alpha I}{c}$$

where $F\;\left[\mathrm{kg}/(s^{2}{\mathrm{cm}}^{2}\right)]$ is the acoustic radiation force, $W_{absorbed\;}\left[W/(100{\;\mathrm{cm}}^{3}\right)]$ is the power absorbed by the medium at a given spatial location, c [m/s] is the sound velocity in the medium, $\alpha \;\left[m^{-1}\right]$ is the absorption coefficient of the medium, and $I\;[W/{\mathrm{cm}}^{2}$] is the temporal average intensity at a given spatial location. In general, the acoustic radiation force also occurs in conventional B-mode ultrasound imaging, but its magnitude is too small to generate any measurable tissue motion [30]. Thus, pushing a tissue using a burst signal with a longer duration (50~100 μs) can generate a localized deformation of the target. These displacements are small, usually less than 20 μm, but they can be detected using a correlation-based method that compares the ultrasound echo signals before and after applying a force. The degree of displacement depends on the magnitude of the force and is inversely proportional to the tissue stiffness [28,29,30,31,32,33,34,35,41].

### 2.2. Relationship between Acoustic Radiation Force Induced Displacement and Internal Pressure

**Figure 1:**
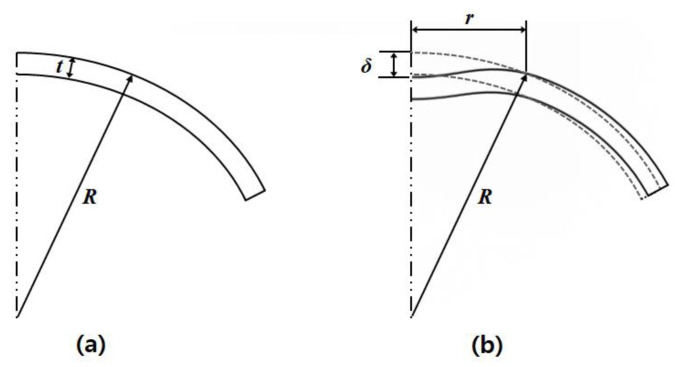
Schematic diagrams of (**a**) undeformed cornea apex and (**b**) deformed shape during application of acoustic radiation force.

In the human eye, the cornea and sclera are constantly stressed by IOP applied to the internal surface of the anterior eye. If an external pressure by acoustic radiation force is applied in the opposite direction to the apex of the cornea, deformation of the cornea will mainly occur radially forward on the apex where acoustic radiation force is overwhelming, as shown in Figure 1 [43,44,45].

**Figure 2:**
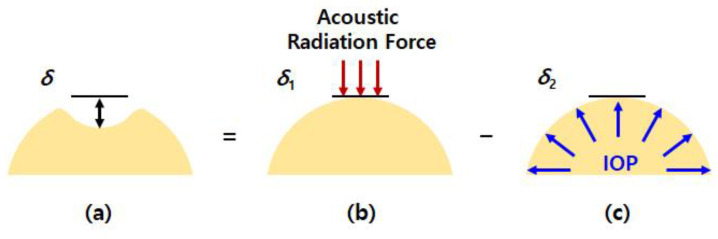
Schematic diagrams of the operational principle of the proposed technique: (**a**) final deformation *δ* is equal to the difference between (**b**) deformation *δ*_1_ by the acoustic radiation force and (**c**) deformation *δ*_2_ by the IOP.

Where, *δ* is a displacement of corneal apex, *r* is a length of acoustic radiation force applied area, *t* is a thickness of cornea, *R* is a radius of anterior cornea. The degree of deformation is affected by the different magnitudes of the IOP and acoustic radiation force acting in the opposite directions. Since two kinds of pressures are simultaneously applied to the an identical area, the final deformation *δ* is equal to the absolute value of the difference between the deformation *δ*_1_ due to the acoustic radiation force and the deformation *δ*_2_ due to the IOP, as expressed by Figure 2 and Equation (2):

(2) $$\delta =\left|\delta_{1}-\delta_{2}\right|$$

Using the ultrasound correlation-based method, the deformation can be measured in a very small micro-scale range. The deformation *δ*_1_ is also generated in the opposite direction of *δ*_2_. In Equation (2), the deformation *δ*_1_ due to the acoustic radiation force on the cornea apex can be determined using the following equation [43,44,45]:

(3) $$\delta_{1}=\frac{\beta \cdot W\cdot \left(R-t/2\right)\sqrt{1-v^{2}}}{Et^{2}}$$

where *W* is the flattening weight concentrated on a small circular area of the cornea, and *β* is a numerical coefficient showing the corneal geometry constant. *R* is the radius of the spherical cornea, *t* is the center thickness of the cornea, *v* is the Poisson’s ratio of the cornea, and *E* is the Young’s modulus of the cornea. The Young’s modulus defines the relationship between the stress and strain of linear elastic materials under a uniaxial deformation so that it represents a mechanical characteristic of the material stiffness. Poisson’s ratio indicates the ratio between lateral and longitudinal strain according to applying a tensile force. For a perfectly incompressible material, Poisson’s ratio is 0.5. Since most soft tissues are nearly incompressible, a Poisson’s ratio value close to 0.5 is commonly used [45]. It was found that the results of tonometry derived from Poisson’s ratio from 0.45 to 0.5 have less than 1% difference, and thus it can be replaced by constant value within the range [46]. In order to determine the value of *β* expressed by Equation (4), *μ* should be obtained through Equation (5) [43,44]. Note that ‘kei’ in Equation (4) indicates the modified Bessel function of the first kind [47]:

(4) $$\beta =\frac{\sqrt{12}}{2\pi }\cdot \mathrm{kei}\cdot \mu$$

(5) $$\mu =r{\left[\frac{12\left(1-v^{2}\right)}{{\left(R-t/2\right)}^{2}t^{2}}\right]}^{1/4}$$

Normally, the coefficient *β* can be determined by using a pre-calculated value of *μ* as shown in Table 1 [43].

In Equation (3), the applanating weight *W* can be obtained by the following equation:

(6) W=ARF_P·A

where *ARF_P* is the applanating pressure produced by the acoustic radiation force, and *A* is the applied area. The value *A* can be obtained by a lateral beam profile since the shape of the deformation pattern is similar to the shape of ultrasound intensity field of the transducer [35]. By substituting Equation (6), Equation (3) can be represented as:

(7) $$\delta_{1}=\frac{\beta \cdot ARF\_P\cdot A\cdot \left(R-t/2\right)\sqrt{1-v^{2}}}{Et^{2}}$$

On the other hand, the deformation *δ*_2_ due to IOP (Figure 2c) on the cornea apex can be calculated using the equation below [46,47,48]:

(8) $$\delta_{2}=\;\frac{IOP\_T\cdot {\left(R-t/2\right)}^{2}\left(1-v\right)}{2Et}$$

where *IOP_T* is the true IOP which we want to measure with the proposed method, *R* is the radius of the spherical cornea, *t* is the corneal thickness, *v* is Poisson’s ratio, and *E* is Young’s modulus of the cornea. Substituting Equations (7) and (8) into Equation (2) gives the following equation:

(9) $$\delta =\left|\delta_{1}-\delta_{2}\right|=\left|\frac{\beta \cdot ARF\_P\cdot A\cdot \left(R-t/2\right)\sqrt{1-v^{2}}}{Et^{2}}-\;\frac{\;IOP\_T\cdot {\left(R-t/2\right)}^{2}\left(1-v\right)}{2Et}\right|$$

Generally, in Equation (9), the final deformation *δ* has a positive value where the external pressure having the same direction as *δ*_1_ is dominant. To represent Equation (9) graphically, we replaced the variables required by the formula with the average values of real human eyes. The average corneal radius *R* and corneal thickness *t* were 7.7 mm and 0.54 mm, respectively, and the Poisson ratio *v* was 0.5 [48,49,50]. By substituting these values into Equation (4) and Table 1, the coefficient *β* was set to 0.362. The pressure-applied area *A* was obtained experimentally from the spatial beam profile. In addition, Young’s modulus (Equation (10)) was defined using a previous finding that proved the relationship between elastic modulus and IOP through mathematical proofs and ocular rigidity test [44]:

(10) E=0.0229·IOP_T

**Figure 3:**
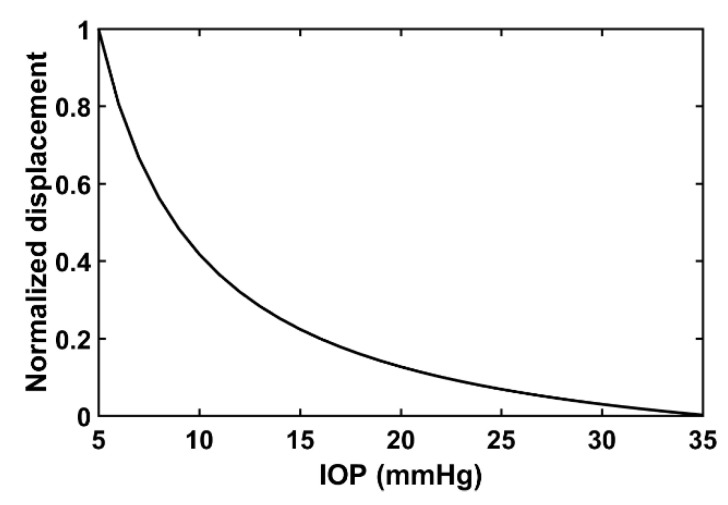
Relationship between normalized displacement obtained by Equation (9) with assumed values and desired IOP range.

Subsequently, since the value *ARF_P*, pressure of acoustic radiation, was not measurable in the current laboratory environment, normalization was conducted to determine the correlation between the axial displacement and IOP. The IOP range to display was set from 5 mmHg to 35 mmHg. Consequently, the normalized displacements expected to occur according to the IOP change can be represented as shown in Figure 3. According to Equation (9) and Figure 3, applying a constant magnitude of acoustic radiation force to a specific corneal area shows that the axial displacement of the cornea varies depending on the IOP of the eye, similar to the form of an exponential function.

When the area to which the acoustic radiation force is applied and the corneal characteristics are the same, the eye with high IOP has a slight deformation in the axial direction, and the eye with low IOP shows a large deformation. In this regard, using the acoustic radiation force can be an alternative scheme to determine corneal deformations for measuring IOP.

## 3. Experimental Validation

### 3.1. Experimental Setup

**Figure 4:**
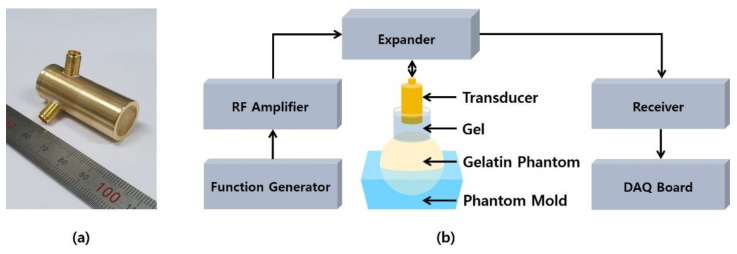
(**a**) Photograph of the 5 MHz prototype ultrasound transducer and (**b**) block diagram of experimental setup using gelatin phantoms with various stiffness values.

A single element transducer with 5 MHz center frequency, 20 mm focal depth, and 2.47 f-number (focal depth/aperture size) was newly fabricated to demonstrate the feasibility of the proposed acoustic radiation force-based IOP measurement method. The aperture of the transducer has a 1–3 composite structure of the press-focused type for improved intensity as shown in Figure 4a. The detailed fabrication process of the prototype transducer is similar to the contents described in [51]. It was driven by in-phase mode to implement the conventional beam shape of the single element transducer [51]. To evaluate the properties of the transducer, electrical impedance measurements and pulse echo tests were performed. In addition, the axial and lateral beam profiles were obtained to calculate the width of pressured area where the acoustic radiation force is applied. A XYZ stage controller (SHOT-304GS, SIGMA KOKI, Tokyo, Japan) was used with a steel wire phantom with 200 μm diameter for measuring the lateral beam profile, and with a quartz reflector for measuring the axial beam profile. Since a hydrophone system was not yet ready, two-way measurement, i.e., pulse-echo response, was conducted to obtain the axial and the lateral beam profiles. Subsequently, it was taken the square root to get one-way response.

**Figure 5:**
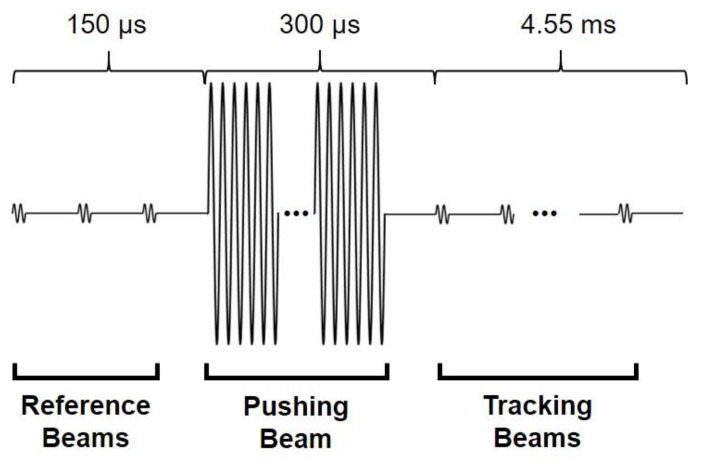
Schematic diagram of the input sequence composed of reference beams, pushing beam, and tracking beams used in experiment. The reference and tracking beams consist of 2-cycle sine wave, and the pushing beam consists of 500-cycle sine wave.

The amplitude mode (A-mode) data was acquired using a customized beam sequence to observe dynamic response of elastic phantom and the change in displacement with elasticity generated by acoustic radiation force. The beam sequence used for these experiments was composed of pushing and tracking beams as shown in Figure 5. In this sequence, three sets of 2-cycle 5-MHz sine wave were transmitted first, which are the tracking beams that serve as a reference for the initial target position. Immediately after the reference beam, a 500-cycle 5-MHz sine wave as the pushing beam was transmitted to generate acoustic pressure and consequently the localized deformation of the target. Multiple tracking beams were then transmitted along the same beam path to observe the temporal response of the target after removal of the acoustic radiation force and determine the axial displacement as a function of time. The amplitude ratio between the pushing and the tracking beams was 10 to 1. A pulse repetition frequency (PRF) of pushing and tracking beams was 20 kHz, and total time duration of the sequence was 5 ms for enough observation of displacement changes over time.

To demonstrate that the degree of displacement varies depending on the stiffness of the target, gelatin-based eye phantoms with different elasticity were made by controlling the concentration of the gelatin powder (Geltech Co. Ltd., Busan, Korea). The various mixing ratios of these phantoms can be referenced in Table 2.

Figure 4b shows the experimental setup to generate and observe the displacement change depending on elasticity of the phantom. The input signal was generated by a function generator (33600A, Keysight Technologies, Santa Clara, CA, USA) and amplified by an amplifier (100A400A, Amplifier Research, Souderton, PA, USA). The amplified signal was applied to the transducer and the reflected echoes were obtained by a receiver (5073PR, Olympus IMS, Waltham, MA, USA) with a 20 dB gain. The received signals were acquired by a data acquisition (DAQ) board (CS121G2, GaGe Applied Technologies Inc., Lachine, QC, Canada). The DAQ board was controlled by a LabVIEW (National Instruments., Austin, TX, USA) software program. Data collection on each phantom was repeated ten times in the same experimental environment.

By using MATLAB (MathWorks Inc., Natick, MA, USA) software, the received radio frequency data was filtered by a band pass finite impulse response (FIR) filter to remove noise and upsampled to 2 GHz using cubic spline interpolation. A one dimensional cross-correlation was calculated in the axial direction between the reference beam and the sequentially acquired tracking beams, and the displacement in the axial direction over time was observed after applying the acoustic radiation force.

### 3.2. Experimental Results

**Figure 6:**
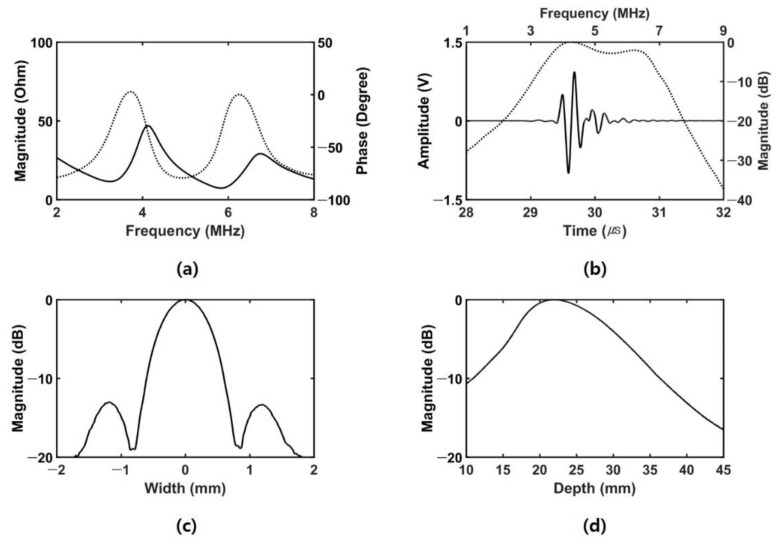
Measured properties of the transducer: (**a**) electrical impedance (solid line: magnitude, dashed line: phase), (**b**) pulse–echo response (solid line: time-domain waveform, dashed line: frequency-domain spectrum), (**c**) lateral beam profile, and (**d**) axial beam profile.

For evaluation of the transducer properties used in this experiment, electrical impedance test, pulse-echo test, and spatial beam profile measurement were conducted as shown in Figure 6.

The electrical impedance was measured using an impedance analyzer (4294A, Keysight, San Jose, CA, USA). The resonance and anti-resonance frequencies were 3.25 MHz (11.44 Ω) and 4.15 MHz (46.82 Ω), respectively, as shown in Figure 6a. Figure 6b shows the pulse-echo response results. The center frequency and -6 dB fractional bandwidth were 5.11 MHz and 67.51%, respectively. The axial and lateral beam profiles were measured using 4-cycle 5-MHz sinusoidal wave. The −6 dB lateral and axial beamwidths were 0.98 mm and 17.1 mm, respectively, as shown in Figure 6c,d.

**Figure 7:**
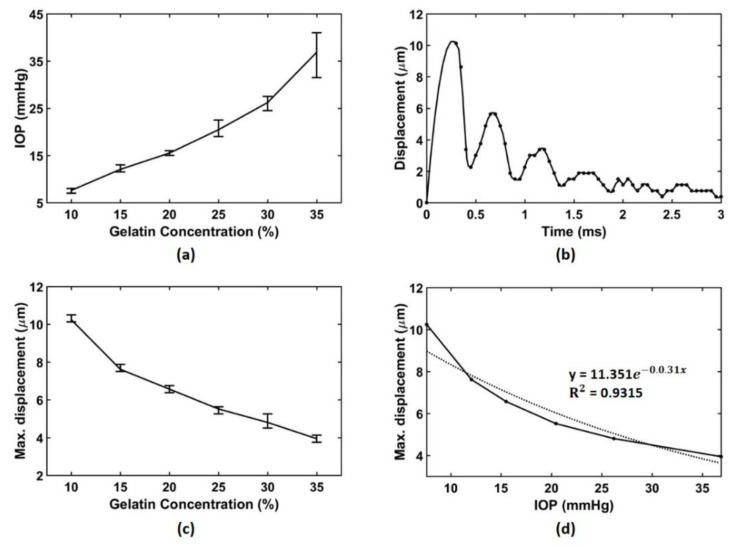
(**a**) Measured IOP values using the rebound tonometer, ten data sets were obtained, (**b**) measured displacement over time in a 10% gelatin phantom, real data (black dot) were interpolated by quadratic spline interpolation method (solid line), (**c**) average of the maximum displacement for each gelatin phantom, measurement was repeated ten times from each phantom, and (**d**) relationship between average of the maximum displacement measured by acoustic radiation force and IOP obtained by the rebound tonometry.

Before conducting the acoustic radiation force experiment, in order to prove that the displacement values to be obtained from our experiments depend on the IOP level, it was necessary to initially confirm that each gelatin phantoms had different IOP characteristics. Accordingly, the IOP values of the gelatin phantom were measured using a commercial rebound tonometer (Ic100, Icare Finland Oy, Vantaa, Finland). Since the Goldmann tonometer, the gold standard for IOP measurement, was not prepared yet, the rebound tonometer, which proved to have a good correlation with the Goldmann tonometer, was selected for comparison [12,17,18,19,21]. The gelatin phantom was fixed to the mold, and the probe tip of the rebound tonometer was placed in the position where the acoustic radiation force is applied. Ten data sets per phantom were recorded, and the measured IOP results for each phantom were shown in Table 3 and Figure 7a.

Figure 7b shows the maximum displacement over time in a 10% gelatin phantom. The black dots indicate the real data, and the solid line represents the interpolated data with quadratic spline interpolation. Although the total time duration of the used sequence was 5 ms, it was enough to observe the displacement change in the phantom within 3 ms. Since the surface of the phantom was stimulated by the acoustic radiation force, the damped vibration was occurred on the surface of the phantom. In the first back and forth oscillation, the maximum amount of movement of the surface was 10.13 μm at 0.3 ms, which can be an indicator of the internal pressure of the phantom. The process of obtaining the displacement over time was repeated ten times on each of the six phantoms, and then the average of the maximum displacements was calculated and plotted in Figure 7c. Table 4 summarizes the measured data based on acoustic radiation force. Assuming that the IOP results by the rebound tonometer in Figure 7a are the true IOP, the correlation between the displacement induced by acoustic radiation force and the IOP can be expressed as shown in Figure 7d. When the IOP and maximum displacement values obtained from six phantoms were matched, the R-squared value of the exponential regression curve was 0.9315 as shown in Figure 7d.

## 4. Discussion and Conclusions

In this study, we demonstrated that an acoustic radiation force capable of generating tissue displacement can be used for IOP measurements. According to the commonly used formulas to calculate the true IOP, it can be seen that the final corneal displacement is derived by calculating the difference between the deformation due to the external force and the deformation due to the internal force of the eye. In an applanation tonometer such as Goldmann tonometer, the final degree of deformation is predetermined to a specific value, and then the IOP is calculated using the force required to deform it to that value. From the same concept, it can be inferred that the final degree of deformation reflects the IOP when a constant force is applied. In this experiment, we tried to verify whether the acoustic radiation force can induce deformation by replacing the applanation force (=external force), and it was confirmed that the displacement value changes according to the IOP.

For this experimental demonstration, we fabricated a 5 MHz ultrasound transducer, and produced eye phantoms with various gelatin concentrations to observe changes in intraocular pressure step by step. The data obtained using the sequence composed of pushing and tracking beams was converted into the axial displacement information over time for each phantom through offline processing. If the stiffness control of fabricated phantoms was conducted properly, the phantoms should show the incrementally changed IOP values as the gelatin concentration increases. As a result of checking the IOPs of the fabricated phantoms with a commercial tonometer as shown in Figure 7a, various IOP values were obtained as we intended, and thus it can be said that the customized phantoms are suitable for use in IOP measurement experiments. As depicted in Figure 7b, it was confirmed that axial displacement of the phantom surface was caused by the acoustic radiation force of the applied pushing beam. Also, after the pushing beam application was completed, the peak deformation was found in the first lobe, and its value gradually decreased over time.

The applied pushing beam is a 500 cycle 5 MHz sine wave, which is ten times larger in amplitude and has a relatively longer time duration compared to the tracking beam. Therefore, the echo caused by the pushing beam was not negligible, and this echo signal makes it difficult for the tracking beam to be transmitted immediately after the pushing beam is terminated. Since both the pushing and the tracking beams of the sequence were composed of 5 MHz sine waves, there was a limit to removing echo signals from the pushing beam by the frequency filtering scheme. Thus, the first tracking beam was transmitted 0.3 ms later after the pushing beam was sent, in this case, the influence of the echo signal of the pushing beam can be minimized. However, if the displacement information can be obtained immediately after the pushing beam, the accurate value closer to the actual displacement can be obtained.

As a result of measuring the IOP values of the six types of gelatin phantoms using the rebound tonometer, it was confirmed that the IOP values were different depending on the concentration. Thus, these gelatin phantoms can be used in experiments to measure IOP values using the acoustic radiation force. The final displacement induced by the acoustic radiation force was dependent on the internal pressure of the object as expected from the formulas, and the R-squared value of the exponential regression curve for those two values was 0.9315, which shows a relatively large goodness to fit.

In this study, it was not possible to directly convert the eye displacement measured in the experiment into IOP due to some limitations. For one thing, the fabricated phantom did not have a thin layer like the actual corneal layer, and we were unable to measure the Young’s modulus and acoustic radiation pressure in our laboratory environment. If the above parameters are ready, it will be possible to get the IOP right away and to compare other conventional procedures. Although our results sufficiently show that the displacement induced by the acoustic radiation force is related to the IOP, further studies including ex-vivo and in-vivo tests will be undertaken to increase the reliability of the proposed technique and investigate the effects of other anatomical confounders.

The main purpose of this study is to validate the method of measuring IOP using displacements generated and tracked by the acoustic radiation force. Therefore, in order to deliver sufficient ultrasound energy, the experiments were performed using a gel between the eye phantom and the ultrasound transducer. However, the use of a gel on a human cornea can cause great discomfort to the patient. Thus, when this technique is applied to a human, the ultrasound transducer should be placed on the eyelid using a gel, and the influence of the eyelid should be removed for precise displacement measurement. In general, the ultrasound acoustic radiation force has been widely used for measuring the elasticity of the organs where the targets are covered by the skin such as breast, thyroid or abdomen [28,30,31,33,35,36]. Thus, the acoustic radiation force can pass through the eyelid and be transmitted to the cornea surface of the human eyes. In addition, since CCT generally affects the measurement results of Goldmann and rebound tonometers, a corneal thickness measurement using an ultrasound based pachymeter should be performed before and after IOP measurement to improve accuracy. However, the proposed acoustic radiation force method has the advantage of being able to measure the corneal thickness at the same time as the IOP measurement.

The reason for using the gelatin phantoms in this study is to ensure that the axial displacement, which can be simultaneously generated and measured by the acoustic radiation force, has correlation with the IOP values or not. In order to achieve this goal, experimental targets with gradually changing IOP values were required, the gelatin phantom capable of changing property was the proper target. Moreover, the gelatin-based phantom is a commonly used target for validation experiments for measuring elasticity or mimicking the structure of corneal tissues [52,53,54]. In this regard, we conducted experiments with the gelatin phantoms for the feasibility validation of our proposed technique. We will try to find a way to gradually change the IOP value of the in-vitro target as the further work. In this experiment, the increase amount in the concentration of the gelatin phantom was determined by considering the range of human IOP. If the error range of the rebound tonometer used in the experiment and the concentration uniformity error of the hand-made gelatin phantom in the laboratory can be reduced, the increase amount in concentration of the gelatin phantom can be more reduced resulting in the increased IOP resolution.

As we mentioned the above, OCE techniques also apply acoustic radiation force to transfer it to the interior of corneal tissues, such as crystalline lens and retinas for elastic imaging [36,37,38,39,40]. Our proposed technique also fires acoustic radiation force to the eyes, but it could have lower force than OCE since it does not need to transfer the force to the inside of eyes and only delivers the force on the surface. In addition, though the acoustic force is also used in the tracking beams for measuring the resulting deformation, it is supposed to cause no problems since it has a short duration and an amplitude equal to one tenth of the pushing beams. Even so, a precise safety test using ex-vivo and in-vivo should be conducted in the further study. 

In this study, the feasibility of the acoustic radiation force-based IOP measurement was verified experimentally. Using the dedicated transducer, customized beam sequence, and gelatin phantoms, we have confirmed that the acoustic radiation force can cause axial deformations on the phantom surface, and this amount of displacement is also related to the IOP. In other words, as the IOP increases, the resistance to the deformation caused by the acoustic radiation force increases and thus the amount of ocular displacement decreases. Conversely, when the IOP decreases, the amount of ocular displacement by the acoustic radiation force increases. Although this study shows that the displacement induced by the acoustic radiation force is related to the IOP, in order to increase the reliability of the proposed method, additional experimental studies such as in-vitro, in-vivo, and sensitivity tests are needed. In addition, since safety issues are very important in the proposed technique, safety tests focused on their effects on corneal endothelial or optic nerve cells are indispensable.

## Figures and Tables

**Table 1:** Calculated *β* given by Equation (3) using *μ* in Equation (4).

*μ*	0	0.1	0.2	0.4	0.6	0.8	1.0	1.2	1.4
*β*	0.433	0.431	0.425	0.408	0.386	0.362	0.337	0.311	0.286

**Table 2:** Gelatin-based phantoms with various different concentration.

	Phantom #1	Phantom #2	Phantom #3	Phantom #4	Phantom #5	Phantom #6
Gelatin (g)	7	10.5	14	17.5	21	24.5
Water (mL)	70	70	70	70	70	70
Concentration (%)	10	15	20	25	30	35

**Table 3:** Measured IOP data using the rebound tonometer.

Concentration	Mean (mmHg)	* SD (mmHg)	Min–Max (mmHg)
Phantom #1 (10%)	7.6	3.4	7–8
Phantom #2 (15%)	12.1	1.8	11.5–13
Phantom #3 (20%)	15.5	3.9	15–16
Phantom #4 (25%)	20.5	3.4	19–22.5
Phantom #5 (30%)	26.2	5.4	24.5–27.5
Phantom #6 (35%)	36.9	7.5	31.5–41

* Standard Deviation.

**Table 4:** Measured displacement induced by the acoustic radiation force.

Concentration	Mean (μm)	* SD (μm)	Min–Max (μm)
Phantom #1 (10%)	10.2	0.2	10.1–10.5
Phantom #2 (15%)	7.6	0.2	7.5–7.9
Phantom #3 (20%)	6.6	0.2	6.4–6.8
Phantom #4 (25%)	5.5	0.2	5.3–5.6
Phantom #5 (30%)	4.8	0.2	4.5–5.3
Phantom #6 (35%)	3.9	0.2	3.8–4.1

* Standard Deviation.

## Data Availability

Not applicable.
